# Inhalational Anesthetics Induce Neuronal Protein Aggregation and Affect ER Trafficking

**DOI:** 10.1038/s41598-018-23335-0

**Published:** 2018-03-27

**Authors:** Matthew Coghlan, Elizabeth Richards, Sadiq Shaik, Pablo Rossi, Ramesh Babu Vanama, Saumel Ahmadi, Christelle Petroz, Mark Crawford, Jason T. Maynes

**Affiliations:** 1 Department of Anesthesia and Pain Medicine, Hospital for Sick Children, Toronto, Canada; 2 Department of Anesthesia, University of Toronto, Toronto, Canada; 3 Program in Molecular Medicine, SickKids Research Institute, Toronto, Canada; 4 Department of Physiology, University of Toronto, Toronto, Canada

## Abstract

Anesthetic agents have been implicated in the causation of neurological and cognitive deficits after surgery, the exacerbation of chronic neurodegenerative disease, and were recently reported to promote the onset of the neurologic respiratory disease Congenital Central Hypoventilation Syndrome (CCHS), related to misfolding of the transcription factor Phox2B. To study how anesthetic agents could affect neuronal function through alterations to protein folding, we created neuronal cell models emulating the graded disease severity of CCHS. We found that the gas anesthetic isoflurane and the opiate morphine potentiated aggregation and mislocalization of Phox2B variants, similar to that seen in CCHS, and observed transcript and protein level changes consistent with activation of the endoplasmic reticulum (ER) unfolded protein response. Attenuation of ER stress pathways did not result in a correction of Phox2B misfolding, indicating a primary effect of isoflurane on protein structure. We also observed that isoflurane hindered the folding and activity of proteins that rely heavily on ER function, like the CFTR channel. Our results show how anesthetic drugs can alter protein folding and induce ER stress, indicating a mechanism by which these agents may affect neuronal function after surgery.

## Introduction

Every year over 60 million anesthetics are administered to patients in the United States, as part of a surgery or for medical procedures^1^. The pharmaceuticals commonly administered during anesthesia, necessarily, modulate neuronal activity, and may have effects that extend beyond the peri-operative period^2^. The adverse effects of anesthetics are more prominent in populations that have a lower capacity to handle stress, like the elderly, where persistent neuronal dysfunction can manifest as memory or cognitive deficits, or the exacerbation of chronic neurodegenerative diseases^3–5^. Post-operative cognitive dysfunction (POCD) develops in ~10–40% of patients, with risk factors including (but not limited to) older age, the occurrence of perioperative complications and pre-existing cerebral/cerebrovascular disease^6^. POCD increases the risk of peri-operative morbidity, mortality and patient use of social assistance^4,6^. The mechanisms by which POCD develops are unknown but may be related to patient physical and psychological stress, immune modulation, or that POCD may occur as an exacerbation of already existing chronic neurologic disease^7,8^. As evidence for the latter, anesthetics were shown to induce tau hyperphosphorylation^9–14^ and amyloid deposition^15,16^ in Alzheimer’s models, dyskinesias in Parkinson’s Disease^17–19^, and huntingtin protein aggregation in models of Huntington’s Disease^20^. Determining how anesthetic drugs may influence POCD or chronic neurologic disease may allow for correction or mitigation, but delineating the contributory role of anesthetics is challenging because of the chronic, generally slow and progressive course of the disease process. A potential window into the effect of anesthetic agents on neurologic disease was illustrated by the reported onset of Congenital Central Hypoventilation Syndrome (CCHS, also known as Ondine’s Curse) after exposure to anesthesia for a routine surgery^21,22^. CCHS is caused by mutations in the transcription factor Phox2B, which normally functions in respiratory control in the brainstem and facilitates respiratory drive^23,24^. Phox2B possesses an unusual stretch of twenty consecutive alanine residues, with unknown function, and CCHS is usually associated with an expansion of this region (up to 35 total alanines). Disease onset shows aggregation of the Phox2B protein in cells of the retrotrapezoid nucleus, causing cytotoxicity and the death of carbon dioxide sensing cells^25,26^. As a result, CCHS patients require tracheostomy and lifelong ventilation. Interestingly, the age of onset and disease severity of CCHS is directly related the number of alanine expansions^27^. With thirteen to fifteen extra alanines (33–35 total), a patient usually requires ventilatory support from birth, but a smaller number of alanines is associated with later disease onset, often occurring after a precipitating event with physiological stress^28,29^. The patient described to have anesthesia-induced onset of CCHS possessed five extra alanines (25 total), and was asymptomatic prior to anesthesia exposure^21^. Non-human disease models and post-mortem pathology reveals that CCHS is associated with Phox2B aggregation in the cytoplasm (as a transcription factor, it is usually nuclear), insinuating that anesthetic agents may be able to alter the folding/aggregation of the protein. This is particularly interesting since many chronic neurodegenerative diseases, at least partially, result from protein misfolding, indicating that anesthesia effects on the folding and aggregation of proteins may be a contributive etiology to POCD. We have utilized the neurologically important context of CCHS to study the effect of anesthetics on protein folding, and have found that the inhalational anesthetic isoflurane is able to induce Phox2B aggregation, upregulate endoplasmic reticulum (ER) stress pathways, and promote misfolding of other clinically important proteins that rely heavily on ER function (i.e. CFTR). Additionally, we found that modulation of ER stress cannot attenuate isoflurane-induced protein misfolding, illustrating a potential direct effect of the anesthetic drug on protein structure, with implications for mitigation or attenuation of POCD.

## Results

### Phox2B alanine expansions emulate CCHS clinical disease presentation

**Figure 1:**
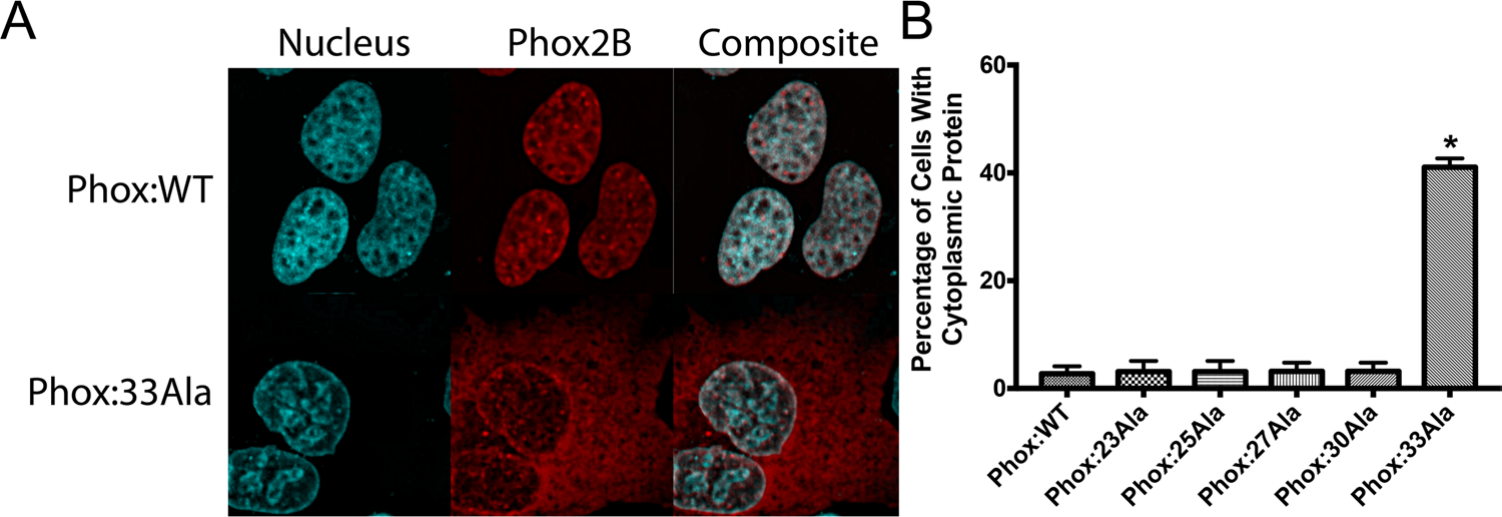
Subcellular localization of Phox2B variants in SH-SY5Y cells. (**A**) The wild-type (20Ala) variant of Phox2B is present almost exclusively in the nucleus, consistent with the protein’s function as a transcription factor. The addition of further alanine residues, representing milder and later-onset versions of CCHS (23-30Ala), are also primarily present in the nucleus. Only the most significant alanine expansion, 33Ala, contains misfolded Phox2B in the cytosol. Nuclear imaging with Hoechst staining and Phox2B via an mCherry N-terminal tag; (**B**) Consistent with previous attempts to express the most significant clinical versions of Phox2B, the addition of 13 extra-alanine residues results in the cytoplasmic localization of the protein in ~50% of cells. At baseline, no other Phox2B variant had a significant number of cells with misfolded, cytoplasmically localized protein (all less than 5%, including the WT protein). Data are presented as mean ± s.e.m. of biological triplicate experiments, where p < 0.05 (denoted by*) was determined using a one-way ANOVA with a Dunnett’s post-hoc test relative to the control (WT) group.

CCHS disease severity is tied to the size of the alanine expansion present in the Phox2B transcription factor^27^. To emulate this clinical observation, we made expression constructs representing the most common patient genotypes, possessing three (23Ala), five (25Ala), seven (27Ala), ten (30Ala) and thirteen (33Ala) extra alanines, plus the wild-type protein (20Ala). All constructs contained an N-terminal mCherry fluorescent tag to facilitate live-cell imaging of protein localization after stable transfection into SH-SY5Y (neuroblastoma) cells (Fig. 1). We observed that the smaller alanine constructs (20Ala, 23Ala, 25Ala, 27Ala and 30Ala) had 97–100% nuclear localization, consistent with the protein being a transcription factor, where the most severe 33Ala variant was present as cytosolic aggregates in approximately half of the cells (41.1% ± 0.9, mean ± s.e.m.) (mCherry alone control vector was present in a uniform cytosolic distribution, without aggregates). These results are consistent with the observed clinical characteristics of CCHS, where patients possessing the 33Ala Phox2B variant have the most severe cellular and physiological phenotype^27^. While systematic alanine-dosing of Phox2B has not been performed previously, earlier experiments observing the cellular localization of 33Ala are consistent with our findings, namely that ~50% of the cells have evidence of cytosolic protein localization^30^.

### Anesthetic agents promote Phox2B aggregation

**Figure 2:**
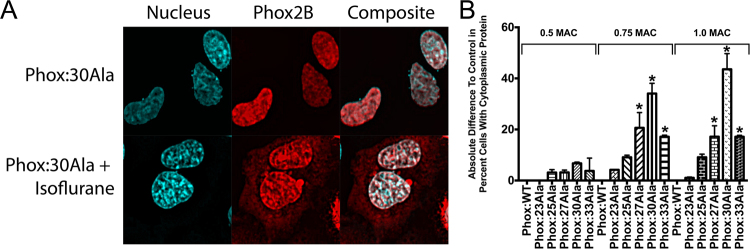
Isoflurane affects Phox2B folding and subcellular localization in SH-SY5Y cells. The addition of isoflurane at 1.1% (1 MAC, usual surgical concentration) for four hours caused Phox2B to misfold and remain in the cytoplasm as aggregates (**A**). The effect was dose-responsive, with respect to both the concentration of isoflurane (0.5, 0.75 and 1.0 MAC) and the number of alanine residues present in the protein (**B**). The effect of isoflurane on Phox2B misfolding was non-significant at the lowest concentration of the gas (0.5 MAC), but increased, and became significant, with higher isoflurane concentrations (0.75 and 1.0 MAC). The response was also dependent on the number of alanines present, becoming significant from 25Ala to 30Ala. The largest expansion (33Ala) also had an increased amount of misfolded protein with cytoplasmic localization, but was less affected than 30Ala, likely because of the baseline severity of the 33Ala variant. Data are presented as mean ± s.e.m. of biological triplicate experiments, where p < 0.05 (denoted by*) was determined using a one-way ANOVA with a Dunnett’s post-hoc test relative to the control (WT) group within that isoflurane dose.

To determine how a surgical anesthesia may initiate the onset of CCHS, we tested pharmaceuticals commonly used in anesthesia for their ability to potentiate Phox2B aggregation and loss of nuclear localization. This included (1) propofol, a hypnotic GABA-A agonist and a general anesthetic, (2) morphine, an opiate analgesic, and (3) isoflurane, the most commonly used inhalational anesthetic and anesthesia maintenance agent, also an agonist of the GABA-A channel. After a four-hour anesthetic drug exposure using clinically-relevant serum concentrations, no agent had any effect on the localization or aggregation of the smaller alanine variants (WT, 23Ala, 25Ala) (Fig. 2, Suppl Figs 1, 2). However, with larger alanine expansions (27Ala and larger), isoflurane began to induce cytoplasmic protein localization and aggregation (Fig. 2). The effect of isoflurane was dose-dependent, showing no statistical difference from control in aggregated, mislocalized protein at 0.5 Minimal Alveolar Concentration (MAC), an intermediate response at 0.75 MAC, and the most significant response at 1.0 MAC (with statistical significance over baseline for the 27Ala, 30Ala and 33Ala variants for both 0.75 and 1.0 MAC, where 1.0 MAC is the anesthetic gas concentration needed to prevent a response to surgical stimulus in one-half of the population, and represents the typical intra-operative amount of anesthetic delivered). For both 0.75 and 1.0 MAC isoflurane, the 30Ala expansion had the most significant change from baseline (34.1% ± 4.0 and 43.6% ± 6.0 more cells with cytosolic localization compared to control, for 0.75 and 1.0 MAC respectively, both p < 0.05). Morphine had a smaller but statistically significant effect on the 30Ala mutation alone, and only at the highest concentration of the drug tested (tested at 1 and 10 μM, 23.3% ± 3.9 more cells with cytosolic localization compared to control for 10 μM, p < 0.05). Interestingly, propofol (tested 0.1 and 1 μM) had no effect on any Phox2B variant, despite binding to the same therapeutic receptor as isoflurane (GABA-A)(Suppl Figs 1, 2). These results indicate that isoflurane most significantly affects Phox2B folding, subcellular localization and aggregation, precipitating the disease phenotype.

### Isoflurane Induces ER Stress Pathways

**Figure 3:**
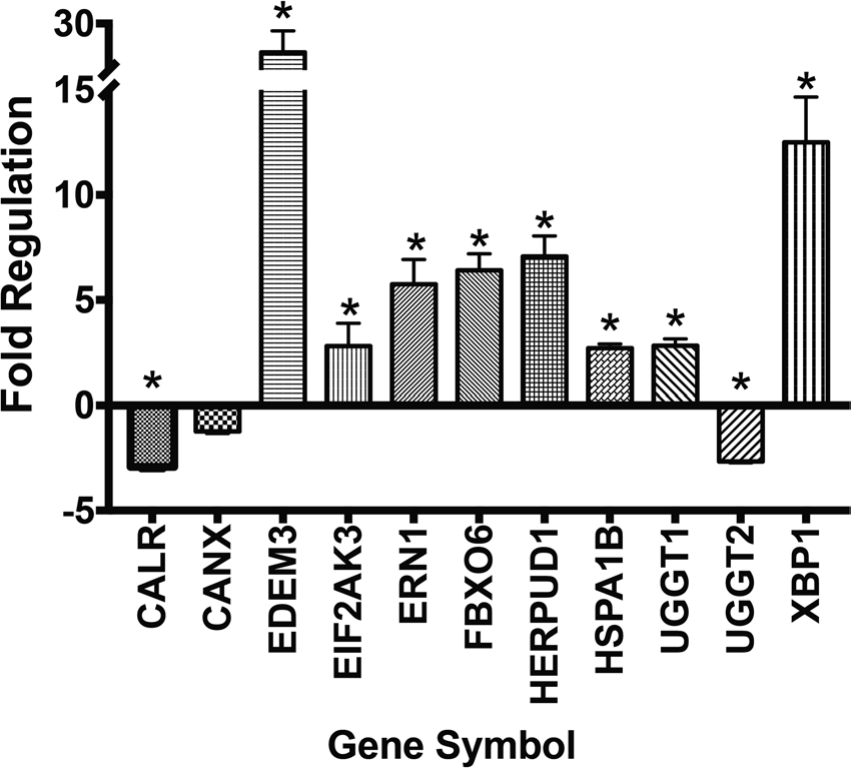
Selected transcript changes with four-hour isoflurane treatment of SH-SY5Y cells. Samples denoted with an asterisk have p < 0.05 after normalization to two internal control (housekeeping) genes (HPRT and GAPDH), with comparison between treated and untreated samples. Data are presented as mean ± s.e.m. of biological triplicate experiments, where p < 0.05 (denoted by*) was determined using a student’s t-test relative to the control group (Analysis using Qiagen GeneGlobe Software).

**Figure 4:**
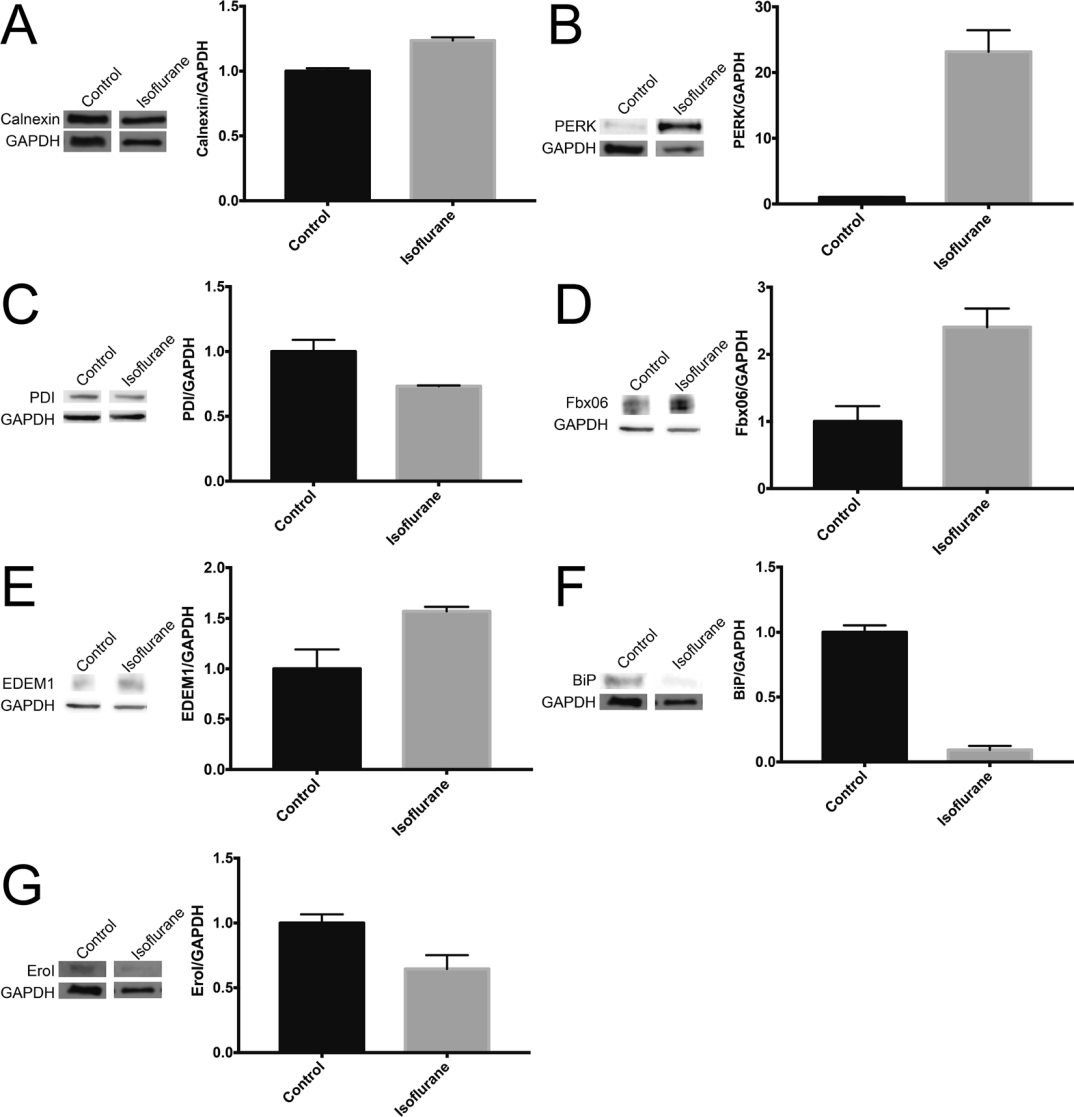
Western blot analysis of proteins involved in ER stress and the UPR in SH-SY5Y cells. With four-hour isoflurane exposure, alterations were seen to (**A**) calnexin, (**B**) PERK, (**C**) protein disulphide isomerase (PDI), (**D**) Fbx06, (**E**) EDEM1, (**F**) BiP and (**G**) EroI. Typical bands shown from biological triplicate experiments. All results were p < 0.05 as determined by student’s t-test between control and isoflurane exposed samples.

In animal models of CCHS, misfolding of Phox2B with cytoplasmic aggregation has been shown to induce cytotoxicity and the death of neurons in the retrotrapezoid nucleus of the brainstem, an area responsible for carbon dioxide sensing^31,32^. To investigate the mechanism by which isoflurane may promote cytotoxicity through protein misfolding, we determined how the anesthetic agent affects ER stress pathways. At the transcript level, we observed that a four-hour exposure of SH-SY5Y cells to 1.0 MAC isoflurane had significant effects on common ER stress pathways affecting translation/gene expression (PERK/EIF2AK3, XBP1, IRE1/ERN1) and protein quality control and folding (calreticulin, ERO1L)(Fig. 3, Suppl Fig. 3 and Table 1). We also observed that isoflurane had a strong and specific effect on the transcript levels for proteins involved in glycoprotein degradation and processing (EDEM1, EDEM3, GANAB, UGGT1) and heat shock proteins/chaperones (DNAJB9, DNAJC3, HSPH1). As a downstream correlation, we investigated how isoflurane altered the level of proteins involved in ER stress pathways and the handling of terminally misfolded proteins (Fig. 4). Similar to the transcript analysis, ER proteins involved in protein folding/disulphide bond formation were significantly altered including calnexin (2.1 ± 0.1 fold increase over control), protein disulphide isomerase (PDI) (1.6 ± 0.1 fold increase) and the oxidoreductase EroI (1.7 ± 0.1 fold decrease). Proteins involved in transcriptional/translational regulation were also affected, including PERK (2.7 ± 0.3 fold increase) and BiP (2.2 ± 0.2 fold decrease), and we saw an increase in enzymes involved in glycoprotein misfolding including EDEM1 (1.5 ± 0.2 fold increase) and FbXO6 (1.7 ± 0.2 fold increase). Therefore, both our transcript- and protein-level analyses indicate that isoflurane can induce activation of ER stress and UPR pathways, with specific emphasis on those involving the handling of terminally misfolded proteins, glycoproteins, and ER-mediated alterations to transcription/translation. These findings are consistent with our observation of isoflurane-induced misfolding and aggregation of Phox2B.

### ER Modulation Reduces Isoflurane-Related ER Stress But Doesn’t Attenuate Protein Misfolding

Our findings do not indicate whether isoflurane has a direct effect on protein folding, leading to activation of the UPR, or if isoflurane-induced ER stress leads to protein aggregation. To help delineate the cause and effect relationship, we utilized small molecules known to modulate protein folding and ER stress. Specifically, we tested the ability of 17-(Allylamino)-17-demethoxygeldanamycin (17-AAG, Hsp90 inhibitor), curcumin (anti-oxidant and anti-protein aggregation natural product) and ibuprofen (previously shown to reduce the aggregation of other poly-Ala expansion proteins) to attenuate the adverse effects of isoflurane^30,33–37^. The first two of these compounds (17AAG and curcumin) have been extensively studied for their anti-cancer benefits, and are of interest for our system since Phox2B variants are also associated with an increased incidence of neuro- and medulloblastoma^38–40^. Additionally, these compounds were illustrated to reduce Phox2B aggregation in a cell model of CCHS (33Ala variant only)^30^. Parental SH-SY5Ycells (no exogenous Phox2B expression, for UPR analysis) and Phox2B cell models (for effects on protein folding) were pre-treated for 48 hours prior to isoflurane exposure (1.0 MAC, four hours), to allow for a full drug effect, and tested for attenuation of ER UPR components involving translational arrest (PERK), oxidative protein folding (EroI), redox sensing and misfolded protein handling (PDI) and unfolded glycoprotein handling (calnexin)^41^. We found that all three compounds were variably able to block/attenuate isoflurane effects on the ER UPR, although only ibuprofen was able to fully modulate all four tested proteins (Suppl Fig. 4). However, none of the agents were able to attenuate isoflurane-induced Phox2B aggregation, even with significant pre-incubation prior to isoflurane exposure. These findings show that the induction of ER stress by isoflurane likely occurs after the initiation of protein misfolding, illustrating how isoflurane may have a direct effect on protein structure.

### Isoflurane Affects the Transport and Activity of CFTR

**Figure 5:**
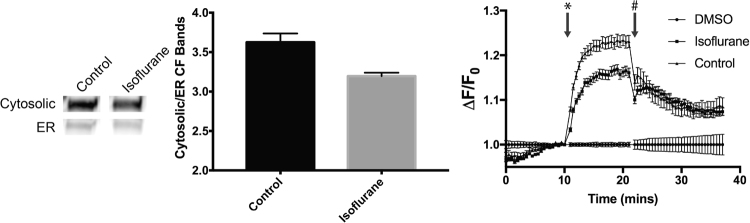
Effect of isoflurane on the trafficking and function of CFTR. Four-hour treatment of HEK cells expressing wild-type CFTR results in a reduction of CFTR glycosylation and trafficking to the cell surface (reduced ratio of cytosolic membrane (band C) to ER (band B) CFTR populations) (left) and a reduction in the chloride channel activity of CFTR (right) (*denotes the addition of CFTR activator, #denotes the addition of CFTR inhibitor). Typical bands shown from biological triplicate experiments. All results were p < 0.05 as determined by student’s t-test between control and isoflurane exposed samples.

**Figure 6:**
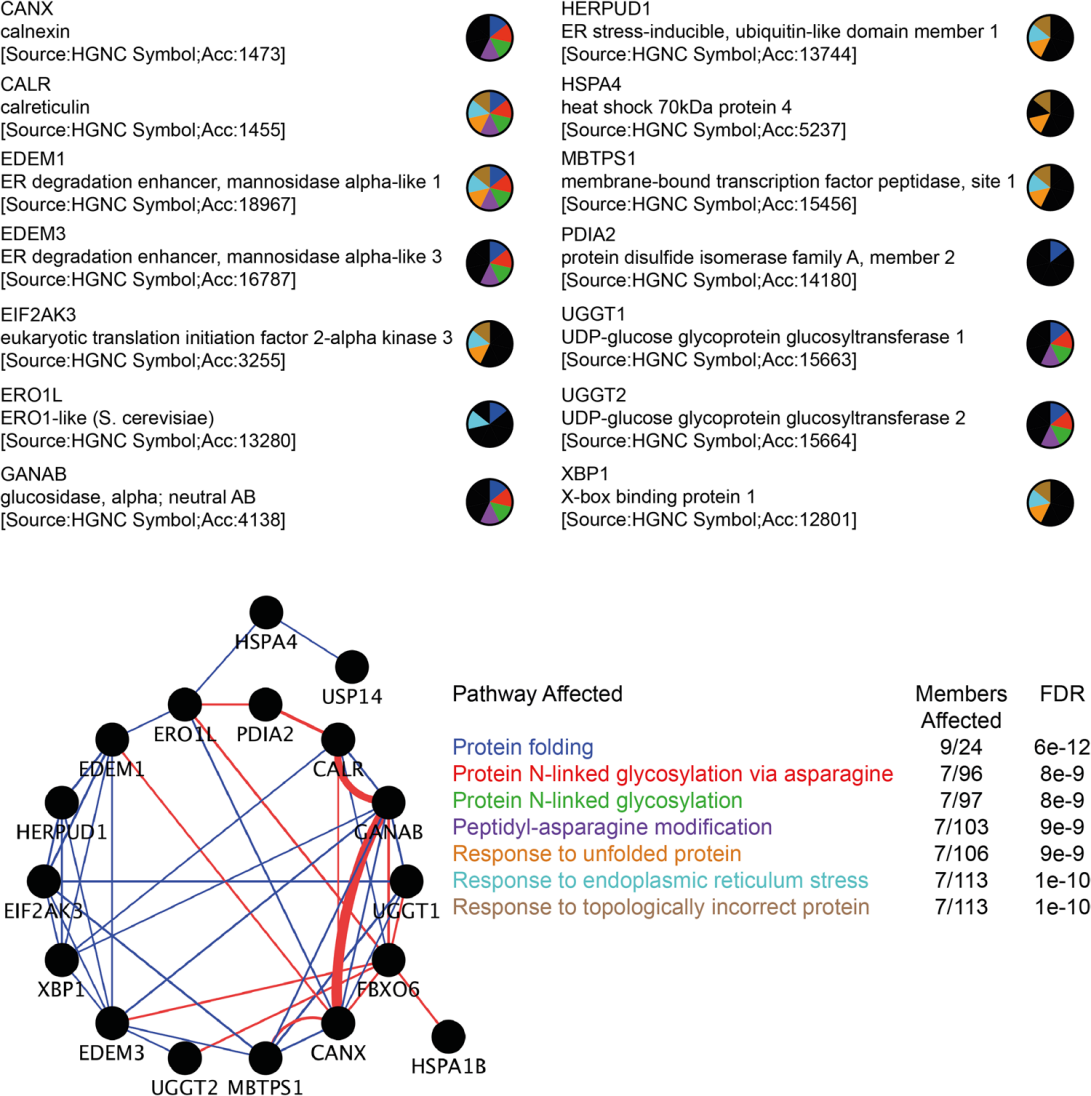
Pathway analysis of isoflurane treated cells. The most significantly affected transcripts were used to determine ER pathways that are affected by isoflurane treatment, highlighting the impact of isoflurane on glycoprotein folding and quality control. Physical (blue) and genetic (red) interactions exist between identified genes and proteins, showing how isoflurane affects protein folding and quality control networks. (Network analysis and FDR performed using the GeneMania software)^58^.

In our transcript and protein level analysis of isoflurane effects on ER stress pathways, we observed an unexpected and specific effect on ER components involved in glycoprotein folding and the handling of terminally misfolded glycoproteins. To determine if isoflurane could have a larger effect on the ability of glycoproteins to fold and function, we utilized a validated cell model of the cystic fibrosis transmembrane conductance regulator (CFTR), a heavily glycosylated protein known to rely significantly on ER function for folding, modification and activity at the cell surface^42^. We found that a four-hour exposure of the cell model to isoflurane (1.0 MAC) reduced cell surface functional expression of the CFTR channel by 24.0% ± 0.3 (p < 0.05) and reduced peak chloride channel flux by 49.6% ± 1.1 (p < 0.05) (Fig. 5). Together, these findings indicate that isoflurane exposure affects the ability of CFTR to fold, transport and perform its regular channel activity, and shows that isoflurane may have a clinically-significant effect on glycosylated protein folding and function (Fig. 6).

## Discussion

The incidence of post-operative cognitive dysfunction is suggested to be 10–40% of elderly patients undergoing non-cardiac surgery, and is associated with increased permanent disability and mortality^3–5^. The high occurrence of the disease in the non-cardiac surgical population is important, as this population has a far lower risk of disease etiology being related directly to surgical factors (i.e. cardiac bypass, microemboli). Thus, the mechanisms involved in POCD involve a combination of patient- and treatment-specific risk factors that can produce a lasting and/or permanent effect on memory and attention. Included in the patient factors is the presence or risk of chronic neurodegenerative diseases^43,44^. In attempting to link anesthesia to the development or exacerbation of chronic neurodegenerative disease, animal models of Alzheimer’s and Parkinson’s disease have shown mixed results, possibly because these diseases are chronic and require years or decades of changes to cellular physiology before overt pathology is observed. Gas anesthetics have been shown to increase certain pathologic indicators (i.e. tau hyperphosphorylation)^10,11,13,15,16^ and induce the aggregation of disease-related proteins (i.e. amyloid and huntington protein)^13,15,16,20^ but causal studies in patients have more mixed results^3,6,7,18,45^.

The report of anesthesia-induced aggregation of a neuronal transcription factor (Phox2B), and the precipitated onset of CCHS, has potentially offered insight into how anesthetic agents can affect neuronal function. We have shown how the anesthetic gas isoflurane can induce Phox2B aggregation at clinically-relevant concentrations in the time span of a normal anesthetic (four hours), and that this aggregation matches what is seen in CCHS patients. Inhaled anesthetic gases were previously shown to alter global protein stability and dynamics^46^, but instead of facilitating unfolding or aggregation, isoflurane was found to increase the thermal stability of albumin. A control protein (myoglobin) was not affected in either dynamics or stability, indicating that protein binding by isoflurane was at least partially specific, and not simply related to the hydrophobicity of the anesthetic. Protein binding specificity was additionally verified by our finding that propofol, another highly hydrophobic anesthetic agent, did not affect Phox2B subcellular localization or aggregation. This also shows that the effect observed for isoflurane cannot be due to action at GABA-A, as both isoflurane and propofol activate this receptor as part of their clinical action.

We could not measure the direct biophysical effect of isoflurane on Phox2B protein stability, as the protein could not be overexpressed recombinantly (likely because the alanine repeat regions are highly hydrophobic, the N-terminal paired-like homeobox region can be expressed in isolation). Previous reports indicate that isoflurane exposure can induce aggregation of huntington protein and amyloid deposition, indicating a primary effect of the gas on protein folding. Our finding that ER-modulating agents could attenuate ER stress pathways, but not affect Phox2B aggregation, agrees with this assertion. The isoflurane effect on Phox2B aggregation far exceeded what we observed with morphine, the latter only affecting the 30Ala variant, and to a smaller degree. Morphine is known to promiscuously bind proteins, including mitochondrial respiratory complexes^47^, but has not been shown to adversely affect protein stability.

A previous study has noted that inhalational anesthetics can induce ER stress and apoptosis in neuronal cells and in fetal mouse brains^48^. This study quantified only CHOP and BiP levels, without exploring more fully the ER effects of gas anesthetics, but did find that the addition of a chemical chaperone reduced ER stress levels. In a separate study using *in vitro* primary neurons, the ER-modulating effect of isoflurane was blocked by dantrolene, implying a ryanodine receptor-mediated mechanism^49^. Isoflurane was also shown to disrupt intracellular calcium homeostasis through actions on the inositol 1, 4, 5-triphosphate receptors^50,51^. Our work expands this analysis, describing isoflurane effects on the ER UPR and cellular transcriptional control, affecting other cellular proteins that heavily rely on ER function (CFTR). Our work implies isoflurane has a much more specific mechanism than just broad calcium dysregulation associated with ryanodine and inositol 1, 4, 5-triphosphate receptor alterations, with gross ER dysfunction and the induction of apoptosis, instead specifically affecting protein folding/aggregation, transcriptional control and the UPR (Fig. 6).

Activation of inflammatory pathways has been causally linked to POCD, and the ER UPR is associated with both the innate immune response and general inflammation pathways^52^. Anesthetic agents have been variably linked to both increases and decreases in inflammation, depending on the tissue and the context of the investigation, although most cases not involving endotoxin or infection-related inflammation indicate that anesthetic gases increase inflammation. Matta *et al*. illustrated that inhalational anesthetics, like isoflurane, can specifically increase neuroinflammatory pathways and increase pain sensation through upregulation of the TRPA1^53^. This effect was direct, as isoflurane was found to bind to and activate TRPA1, and was more significant for isoflurane over other anesthetics. In other clinical studies, gas anesthetics were found to increase inflammation in patients undergoing hysterectomy^54^ and cholecystectomy^55^ and gas anesthetics have been associated with lung inflammation and a reduction in post-operative lung function^56^, interesting in the context of our finding of reduced CFTR function and translocation. Alterations to PERK have been postulated as a link between the UPR, inflammation and chronic neurodegenerative disease, as inactivation of PERK was shown to reduce inflammation in models of Alzheimer’s^57^. We found significant alterations to PERK transcript and protein levels after isoflurane exposure, perhaps providing a causal link between anesthesia, ER stress and chronic neurodegenerative disease.

In summary, we have shown how the neuronal transcription factor Phox2B is affected by anesthetic agents, most significantly isoflurane, and shown how anesthesia could potentially precipitate the onset of CCHS. Isoflurane induces aggregation of Phox2B, inducing ER stress and activating the ER UPR, including pathways involved in glycoprotein handling (with alterations to CFTR trafficking and function). Our results show how anesthetic agents could precipitate or exacerbate POCD and chronic neurological diseases through ER stress. We found that agents known to promote ER function were able to attenuate isoflurane-induced ER stress pathways, but were unable to eliminate the inciting incident, namely protein misfolding/aggregation. Our work suggests a mechanism and a potential mitigating strategy for the neurological and cognitive effects of anesthesia in at risk-populations, to reduce the incidence of POCD and the exacerbation of chronic neurodegenerative diseases like Alzheimer’s and Parkinson’s.

## Methods

### Creation of Phox2B Constructs

Wild-type Phox2B sequence was taken from NCBI (NP_003915.2). Additional alanine residues were added by mutagenic PCR by successively adding codons to complementary primers (QuikChange Mutagenesis, Agilent). At each step, either two or three additional alanine codons were present in the mutagenic primers to gradually build the 23Ala, 25Ala, 27Ala, 30Ala and 33Ala constructs. Attempts to add more than two or three codons at a single step were unsuccessful, presumably because of the high GC content of alanine codons and the resulting high melting temperature of the mutagenic primers. The DNA sequence of the added codons was derived from the Phox2B sequence poly-alanine region, to maintain the codon bias that exists in the endogenous coding sequence. After DNA sequence verification, each of the coding sequences was placed into the pDONR201 Gateway Entry Vector (ThermoFisher) using primers containing the attB1 and attB2 recombination sites, and then moved to a Gateway Destination pcDNA3.1 mammalian expression vector with an N-terminal mCherry tag using standard Gateway Clonase methodology. Each construct was transfected into SH-SY5Y cells using the Effectene chemical transfection reagent (Qiagen). Positive clones were selected using the G418 antibiotic (300 μg/mL) over a period of three weeks. Cells were stained with Hoechst33342 (Invitrogen) to identify nuclei, before live-cell imaging. Cells were imaged using an Olympus IX81 confocal microscope with a 40X objective lens and a C900-13 EM CCD (Hamamatsu Photonics), with Volocity Software (Perkin-Elmer). Protein localization was determined by calculating the overlap of mCherry-Phox2B with the nuclear Hoechst33342 staining, in an automated and unbiased manner using ImageJ (NIH). A Gaussian filter was applied to each color channel of the images (blue for nuclei, red for mCherry-linked Phox2B), with automated segmentation and calculation of a Pearson Coefficient for each cell (object). A Pearson coefficient of <0.5 between color channels was taken to represent cytosolic localization of Phox2B (significant Phox2B outside the nucleus). Using this procedure, a minimum of 300 cells per condition, in biological triplicates, was analyzed.

### Anesthesia and Drug Exposures

Gas anesthetic exposures were performed using a custom-built Plexiglas chamber placed inside a standard cell culture incubator for temperature control. The chamber received gas from a cylinder containing a mixture of 21% oxygen/5% carbon dioxide/74% nitrogen. An inline isoflurane vaporizer (Cyprane) was used to maintain the required concentration of anesthetic gas in the carrier. An exit port on the chamber sampled gas continuously to a gas analyzer (Datex-Ohmeda Capnomac), to ensure the concentration of isoflurane, oxygen and carbon dioxide remained constant throughout the experiment. The system was equilibrated at the desired isoflurane concentration and temperature (37° Celsius) before placing cells in minimal media (DMEM) inside the chamber. For non-gas anesthetic exposures, propofol (pure, no intralipid present) or morphine (preservative free)(both Sigma-Aldrich) were added to fresh minimal media at the desired concentration, and pH (with 5% CO_2_) and temperature (37° Celsius) equilibrated before being placed on the cells. For experiments involving chemical modifiers of the UPR and misfolded protein handling, 17-(Allyloamino)-17-demethoxygeldanamycin (17-AAG) (Sigma-Aldrich) was prepared at 1 mM stock solution in DMSO and diluted in fresh complete media (DMEM +10% FBS) to a final concentration of 100 nM, curcumin (Sigma-Aldrich) was prepared at 30 mM stock solution in ethanol and diluted in fresh complete media to a final concentration of 8 μM, and ibuprofen (Sigma) was prepared at 50 mM stock solution in water and diluted in fresh complete media to a final concentration of 10 μM. Therefore, the final concentration of DMSO or ethanol in any experiment was less than 0.1%. In each case, cells were treated with one of 17-AAG/curcumin/ibuprofen for 48 hours prior to imaging or cell lysate collection for Western blot analysis. Complete media was utilized because of the extended incubation of cells to the chemical modifiers (48 hours), avoiding any serum starvation effects.

### Western Blot Analysis

Cells were harvested by scraping, before lysis in RIPA buffer (BioBasic) and clarification by centrifugation. Total cell lysates (40 μg) were separated using SDS-PAGE (20%, w/v) and transferred to a 0.2 um PVDF membrane with the Bio-Rad Trans-Blot Turbo Transfer System. The membrane was blocked for one hour with 5% skim milk in TBST buffer, followed by overnight incubation with the primary antibody in TBST at 4° Celsius. The membrane was washed three times with TBST before incubation with a species-specific horseradish peroxidase-linked secondary antibody for one hour at room temperature. Protein levels were determined using the Enhanced Chemiluinescence System (GE Healthcare) scanned on the BioRad Gel-Doc XRSystem (Bio-Rad). Antibodies used: anti-EroI, -calnexin, -PDI, -PERK and -BiP antibodies were obtained as part of the ER Stress Antibody Kit (Cell Signaling, cat. 9956 T), anti-EDEM1 (Abcam, cat. ab200645), anti-Fbx06 (Abcam, cat. ab57058), anti-human CFTR NBD2-specific (amino acids 1204–1211) murine monoclonal antibody 596 (1:30,000, University of North Carolina at Chapel Hill, Chapel Hill, NC, Courtesy Cystic Fibrosis Foundation Therapeutics){CHIBAFALEK:1999ey}. Secondary antibodies included Goat Anti-Mouse IgG (H + L)-HRP (Biorad cat. 1721011) and Goat Anti-Rabbit IgG (H + L)-HRP (Biorad cat. 1721019).

### Quantitative PCR Analysis

Total RNA was isolated from SH-SY5Y neurons using Qiagen’s RNeasy Mini Kit (cat. 74104), with first strand cDNA synthesis using Qiagen’s RT2 First Strand Kit (cat. 330401). Changes in ER stress-related transcript levels were analyzed using the RT2 Profiler PCR Array for the Unfolded Protein Response (Qiagen cat. PAHS-089Z). qPCR analysis was performed using the C100 Touch Thermal Cycler-CFX96 Real Time PCR (Bio-Rad). All of the above was performed as per the manufacturer’s instructions. Analysis of results, including significance of change, was performed using the GeneGlobe Software (Qiagen). Network analysis was performed using GeneMania^58^. All results are the result of three biological replicates.

### CFTR Function and Localization Assays

The creation of HEK cells expressing wild-type CFTR was described previously^42^. The CFTR functional assay was performed as previously described^59^. Briefly, cells were read in black, clear bottom plates (Corning) in a fluorescence plate reader (SpectraMax i3; Molecular Devices) at 37 °C. After recording a baseline fluorescence (excitation: 530 nm, emission: 560 nm), CFTR was stimulated using the cAMP agonist forskolin (10 μM; Sigma) with DMSO vehicle used as a negative control. CFTR-mediated depolarization of the plasma membrane was detected as an increase in fluorescence. The assay was terminated by the addition of the CFTR inhibitor CFTRinh-172 (10 μM; Cystic Fibrosis Foundation Therapeutics). Changes in transmembrane potential were normalized to the measurement taken at the time of agonist (i.e., DMSO, or forskolin) addition. CFTR trafficking (ER vs plasma membrane) was determined by quantifying the ratio of the mature cytosolic membrane protein (“band C”) to the immature ER-localized form of the protein (“band B”), using the Western blotting methods described above^60^.

### Statistical Analysis

All graphs were created and statistical analysis performed using Prism v7.0 (Graphpad Software). For protein localization and Western Blot experiments, data are represented as mean ± standard error of the mean (s.e.m). With the exception of the gene expression and network analyses (which are described above), statistical significance was defined as p < 0.05 and was calculated using a one-way ANOVA with a Dunnett’s post-hoc test relative to the control (WT) group.

### Data availability

The datasets generated during and/or analysed during the current study are available from the corresponding author on reasonable request.

## Electronic supplementary material

Supplementary Figures
