# Supplementary material for: Inhalational Anesthetics Induce Neuronal Protein Aggregation and Affect ER Trafficking
Source: Sci Rep. 2018 Mar 27;8:5275. doi: 10.1038/s41598-018-23335-0 (PMC5869676; doi:10.1038/s41598-018-23335-0)
Supplement: Supplementary file 1 — Supplementary Figures [file 41598_2018_23335_MOESM1_ESM.pdf]

Inhalational Anesthetics Induce Neuronal Protein Aggregation and Affect ER Trafficking

Matthew Coghlan, MD[1], Elizabeth Richards, MD[1], Sadiq Shaik, MD[1], Pablo Rossi, MD[1], Ramesh Babu Vanama, PhD[1], Saumel Ahmadi, MD[3,4], Christelle Petroz[1], Mark Crawford, M.B.B.S[1,2], Jason T Maynes, PhD/MD[1,2,3]

[1]Department of Anesthesia and Pain Medicine, Hospital for Sick Children, Toronto, Canada
[2]Department of Anesthesia, University of Toronto, Toronto, Canada
[3]Division of Molecular Medicine, SickKids Research Institute, Toronto, Canada.
[4]Department of Physiology, University of Toronto, Toronto, Canada.

Correspondence: Jason T Maynes, PhD/MD
Department of Anesthesia and Pain Medicine
Hospital for Sick Children
555 University Ave.
Toronto, ON, M5G 1X8
Canada
Tel: 416-813-5934
E-mail: jason.maynes@sickkids.ca

SUPPLEMENTARY FIGURES

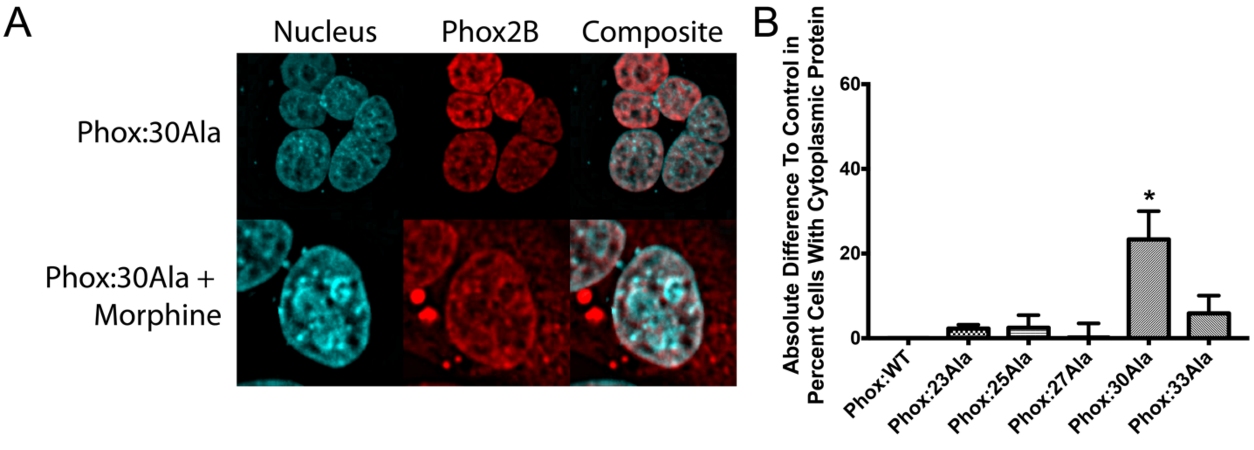

Supplementary Figure 1. Morphine effects on Phox2B folding and subcellular localization in SH-SY5Y cells. (A) Morphine (10 μM but not 1 μM) was able to potentiate aggregation of only the most metastable version of Phox2B, the Phox:30Ala variant, however (B) the severity of the misfolding was less than cells exposed to isoflurane. Only the 30Ala variant is statistically different from baseline ($p < 0.05$). Data are presented as mean ± s.e.m. of biological triplicate experiments, where $p < 0.05$ (denoted by *) was determined using a one-way ANOVA with a Dunnett's post-hoc test relative to the control (WT) group.

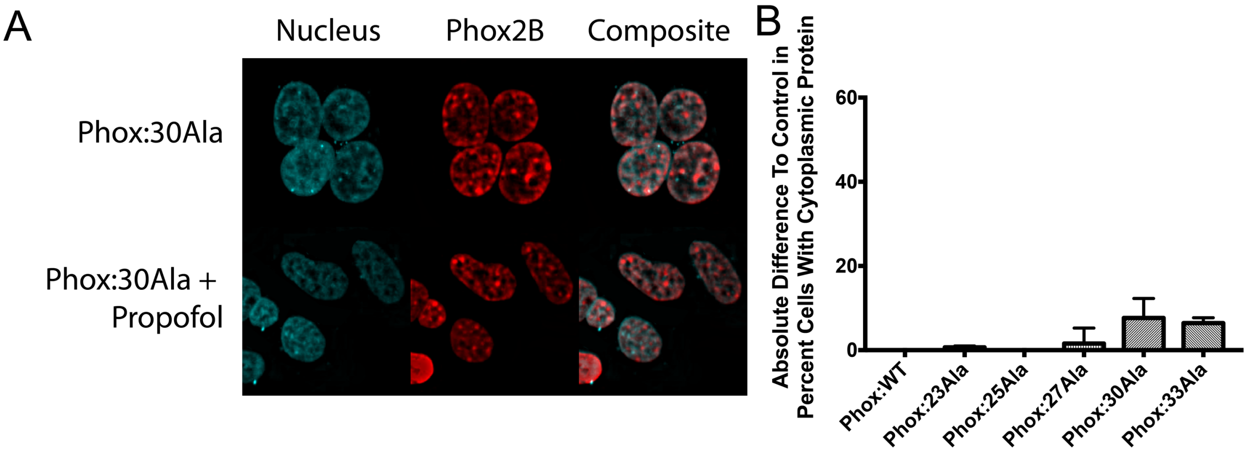

Supplementary Figure 2. Propofol effects on Phox2B folding and subcellular localization in SH-SY5Y cells. Propofol (1 or 10 μM) was unable to potentiate Phox2B aggregation for any variant. No values are statistically different than baseline. Data are presented as mean ± s.e.m. of biological triplicate experiments, where $p < 0.05$ (denoted by *) was determined using a one-way ANOVA with a Dunnett's post-hoc test relative to the control (WT) group.

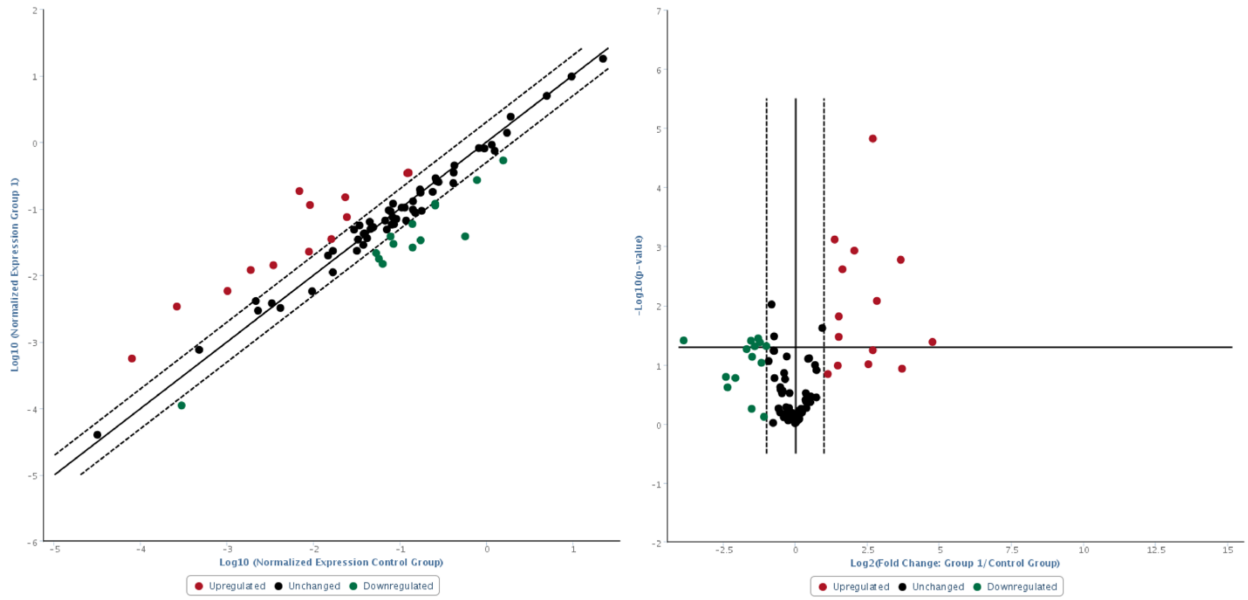

Supplementary Figure 3. Scatter (left) and volcano (right) plots from qPCR analysis of cells exposed to isoflurane for four hours. Statistically up-regulated genes are in red and down-regulated in green, with p-values and fold-change levels in Supplementary table 1.

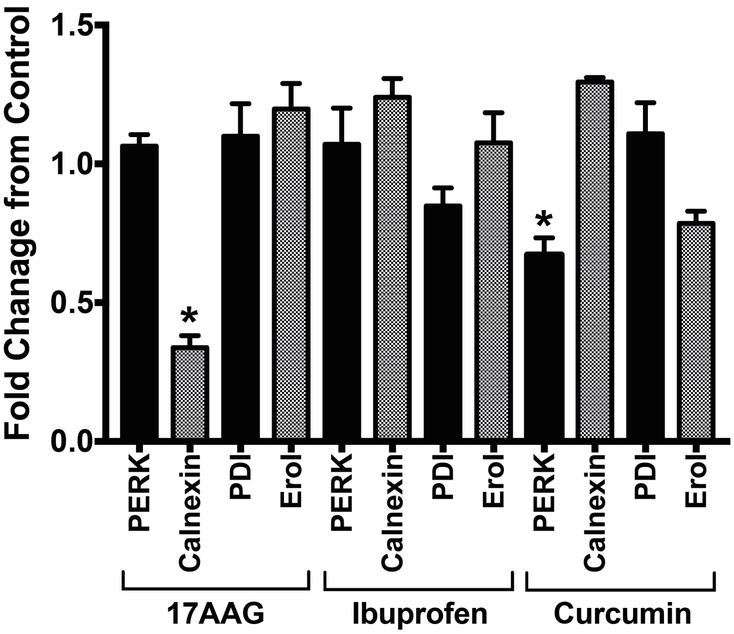

Supplementary Figure 4. Chemical modifiers of ER stress and protein aggregation are able to attenuate isoflurane-induced changes to UPR markers. With cellular preincubation (48 hours), each agent (17AAG (100 nM), ibuprofen (10 μM) and curcumin (8 μM)) was able to significantly attenuate ER stress and UPR activation by isoflurane, but only ibuprofen was able to universally correct all markers. Data are presented as mean ± s.e.m. of biological triplicate experiments, where $p < 0.05$ (denoted by *) was determined using a one-way ANOVA with a Dunnett's post-hoc test relative to the control (unexposed) group.

Supplementary Table 1. RT-PCR values for transcripts involved in ER stress, the ER unfolded protein response (UPR) and ER-stress related signaling pathways. Transcripts denoted with ">>" were not present in control samples, and only had detectable levels in isoflurane exposed samples.

**Supplementary Table 1. RT-PCR Values from Cells Exposed to Isoflurane**		
**Gene**	**Fold Change**	**p-Value**
AMFR	-2.0246	0.048621

ATF6B	-2.4596	0.035985
CALR	-2.9276	0.039494
EDEM1	>>	>>
EDEM3	26.9184	0.041386
EIF2AK3	2.8187	0.033713
ERO1L	>>	>>
FBXO6	6.4176	0.000015
GANAB	>>	>>
HERPUD1	7.0712	0.008361
HSPA1B	2.5627	0.000767
HSPA4	3.0869	0.002438
MANF	-2.3582	0.041659
MBTPS1	-14.7563	0.03902
PRKCSH	-1.6787	0.033108
RNF139	-1.7814	0.009561
UBE2J2	1.9041	0.02407
UGGT1	2.8229	0.015265
UGGT2	-2.6768	0.048982
USP14	4.1111	0.001182
XBP1	12.5289	0.001685
